# Efficacy of Acupuncture for Bell’s Palsy: A Systematic Review and Meta-Analysis of Randomized Controlled Trials

**DOI:** 10.1371/journal.pone.0121880

**Published:** 2015-05-14

**Authors:** Pingping Li, Tangmeng Qiu, Chao Qin

**Affiliations:** Department of Neurology, First Affiliated Hospital, Guangxi Medical University, Nanning, Guangxi, China; Cardiff University, UNITED KINGDOM

## Abstract

Acupuncture has emerged as an alternative therapy for Bell’s palsy in both adults and children. However, the use of acupuncture is controversial. We conducted a systematic review and meta-analysis to assess the efficacy of acupuncture for Bell’s palsy. We searched PubMed, Embase, and the Cochrane Central Register of Controlled Trials, irrespective of any language restrictions. Randomized controlled trials comparing acupuncture with other therapies for Bell’s palsy in adults or children were included. Fourteen randomized controlled trials involving 1541 individuals were included in this meta-analysis. Significant association was observed in acupuncture with a higher effective response rate for Bell’s palsy (relative risk, 1.14; 95% confidence interval, 1.04–1.25; *P* = 0.005) but there was a heterogeneity among the studies (*I* ^2^ = 87%). An assessment of the included studies revealed a high risk of bias in methodological quality. An evaluation of the incidence of complications was not available, owing to incomplete data. Acupuncture seems to be an effective therapy for Bell’s palsy, but there was insufficient evidence to support the efficacy and safety of acupuncture. However, the results should be interpreted cautiously, because of the poor quality and heterogeneity of the included studies.

## Introduction

Bell’s palsy is an acute, ipsilateral facial nerve paralysis with unknown etiology, which can result in weakness of the muscles of facial expression[1]. The clinical features of Bell’s palsy may include sudden onset, unilateral, weakness of the facial nerve, auricular pain, and headaches. Moreover, retroauricular pain may lead to impaired tolerance of noise and ipsilateral disturbance of taste[2]. Bell’s palsy is the most common dysfunction affecting the facial nerves[3]; it affects 11–40 persons per 100,000 population each year[4] and 1 in 60 suffers a lifetime risk[5]. More than 60,000 persons were affected by this disease each year in the United States alone[6]. Most patients with Bell’s palsy recover normally within 3 weeks, with or without medical intervention[7]. However, full restoration may take up to 9 months in some cases and up to 30% of patients are left with complications, such as potentially disfiguring facial weakness or persistent lacrimation, needing further medical therapy[8–10].

Most cases of Bell’s palsy recover spontaneously. Therefore, medical interventions aim to promote the recovery process and minimize the risk of complications and long-term effects. Management includes eye protection, treatment with corticosteroids or antivirals, physical therapy, surgery, and acupuncture. Eye ointment is used widely to avoid trauma to and drying out of the cornea. Corticosteroids have long been used in Bell’s palsy, thanks to their powerful anti-inflammatory effect, and have been proven to be an effective treatment. Previous studies showed evidence for the presence of the herpes simplex virus in some cases of Bell’s palsy[11–15]. Thus, antiviral agents were applied in some cases. As optional treatments of Bell’s palsy, no particular benefits of physical therapy or surgical operation have been reported. Acupuncture is known to be safe for Bell’s palsy[16] and no evidence of harm has been reported so far. A Cochrane review on the efficacy of acupuncture for Bell’s palsy was unable to draw conclusions successfully, owing to defects in experimental design and reports of the included studies[17]. Recently, more studies on the topic have been published, and the results remain conflicting. Therefore, we have conducted this updated systematic review and meta-analysis of randomized controlled trials (RCTs) to compare the efficacy of acupuncture with other therapies in Bell’s palsy.

## Materials and Methods

This systematic review and meta-analysis were performed in adherence to the Preferred Reporting Items for Systematic Reviews and Meta-Analyses (PRISMA)[18].

### Search strategy and study selection

The following databases were searched by two investigators (Li P and Qiu T) independently (from inception to July 2014): PubMed, Embase, and the Cochrane Register of Controlled Trials. We searched terms related to Bell’s palsy (including a Medical Subject Headings (MeSH) search using ‘Bell’s palsy’ and a keyword search using ‘Bell’s palsy’, ‘facial paralysis’, and ‘idiopathic facial paralysis’) and terms related to acupuncture (including a MeSH search using ‘acupuncture’ and ‘acupuncture therapy’ and a keyword search using ‘acupuncture’). The reference lists of the screened full-text studies were searched manually to find potentially relevant studies.

Studies were included if they fulfilled the following selection criteria: [1] study design: RCTs; [2] population: patients with Bell’s palsy; [3] intervention: acupuncture technique that was limited to conventional stimulation of points by needle insertion and electric acupuncture stimulation; [4] comparison: other intervention protocols.

Disagreements about selection criteria were solved by discussion of all the review authors.

### Data extraction and outcome measures

The following information was extracted independently by two investigators (Li P and Qiu T): first author, number of cases and controls, country, ethnicity, mean age, experience of operators. All these data were recorded in a form. Disagreements were resolved by consensus. Two studies were found to be trial protocols without result data. Missing data were obtained by contacting corresponding authors whenever possible. The correspondence author of one study, Professor Zhao[19], informed us that the trial could be completed by the end of this year; thus there was no available data. No reply was received from Kwon *et al*[20].

The primary outcome was the number of participants with complete or partial recovery of facial neural function measured by total effective rate of patients after acupuncture therapy. Effective was identified as the facial paralysis was complete or partial recovered after acupuncture therapy. Total effective rate was calculated by the proportion of participants with effective outcome. Secondary outcome measures included mean therapy time, skills with acupuncture, adverse effects, and the incidence of complications during manipulation. As described above, therapy time was calculated by minute, and the degree of skills of the operator was identified as skilled or not skilled. Adverse effects and complications included infection, needlesickness, needle breakage and hematoma.

### Assessment of bias risks and methodological quality of included studies

The following aspects of each included study were evaluated independently by two investigators (Li P and Qiu T): random sequence generation; allocation concealment; blinding of participants and personnel; blinding of outcome assessment; incomplete outcome data; selective reporting; other bias. A value of ‘high’, ‘low’, or ‘unclear’ was given for each item. The assessment scheme complies with guidelines outlined in the Cochrane handbook for systematic reviews of interventions (version 5.1.0)[21].

### Statistical analysis

Relative risks (RR) with 95% confidence intervals (CI) were evaluated for dichotomous outcomes. Fixed effects or random effects were used, depending on the existence of heterogeneity. Heterogeneity was assessed using the *I* ^2^ statistic. Significant heterogeneity existed for *I* ^2^ > 50%[22]. When heterogeneity was observed, a sensitivity analysis was conducted by excluding one article each time. Subgroup analysis was carried out by type of therapy and methodological quality of the included studies. Potential publication bias was investigated using Begg funnel plots. Begg and Egger tests were also conducted to explore possible publication bias[23, 24]. A value of *P* less than 0.05 was considered to be statistically significant. All available data were analyzed using RevMan 5.3 (The Nordic Cochrane Centre, Copenhagen, Denmark) and Stata 12.0 (Stata Corporation, College Station, TX, USA).

## Results

### Study selection and characteristics

**Fig 1:**
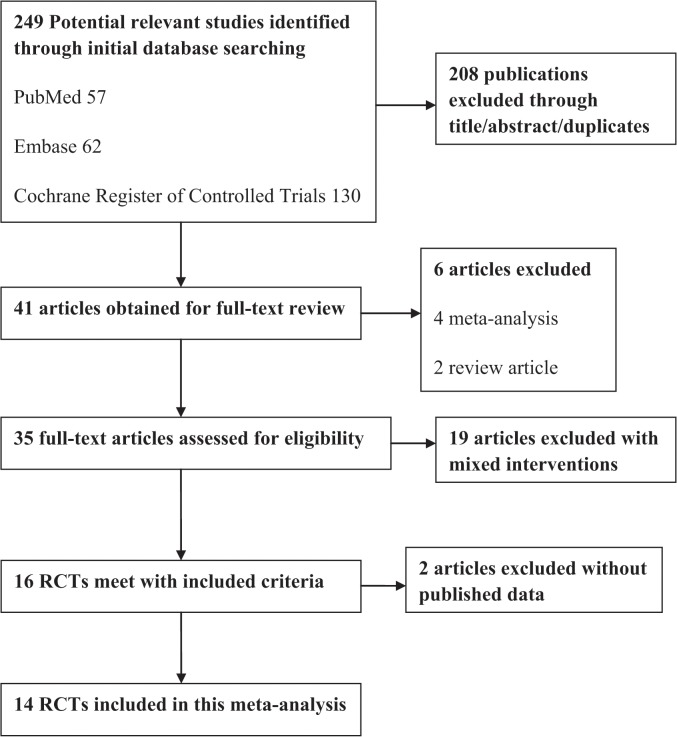
Flowchart of study search.

A flowchart of search selection and results is shown in Fig 1. We identified 249 potentially relevant articles, of which only the full texts of 41 publications were obtained for further review. Six articles were excluded as they were not RCTs: three meta-analyses[17, 25, 26] and three reviews. A total of 19 studies with mixed interventions did not meet our inclusion criteria and were excluded: eight studies compared different types of the effectiveness of acupuncture[27–34]; nine studies investigated acupuncture versus other traditional Chinese medical therapies (one compared acupuncture against blood-letting[35], one compared acupuncture against cupping[36], one studied acupuncture against herbal acupoint sticking[37], one studied acupuncture combined manipulation against acupuncture[38], one investigated the effectiveness of stellate ganglion block plus acupuncture versus acupuncture alone[39], and four articles compared acupuncture against or in combination with moxibustion[40–43]); two studies[31, 39] were replicates of previous articles[44, 45]. Therefore, of the 41 full-text publications obtained, only 16 RCTs[19, 20, 46–59] met our inclusion criteria. Of the 16 included RCTs, two were protocols without published result data[19, 20], thus were excluded. Finially, a total of 14 studies were included in this meta-analysis. Characteristics of the 14 included RCTs are shown in Table 1. All these included studies were conducted in China. Thirteen studies were written in Chinese and one in English[48]. A total of 1541 cases were enrolled in this meta-analysis, with sample sizes ranging from 39 to 320. The participants were randomly assigned to acupuncture group or control group, with their mean age ranging from 6 to 95. The average time from onset for patients with Bell’s palsy ranges from 1 day to 6 months. Eleven studies compared acupuncture with other/no interventions: eight RCTs used drug therapy as a control, one used electrotherapeutic apparatus[49], one used Chinese traditional manipulation[46], one RCT used stellate ganglion block therapy as a control[50]. In addition, five studies[51, 54, 56–58] compared the efficacy of acupuncture plus drug therapy against drug therapy. Of these included RCTs, five studies[46, 49–52] used electroacupuncture techniques while 9 studies chose conventional acupuncture. A total of 11 studies used response rate as an outcome measure. House–Brackmann scoring was used in three studies[48, 54, 57], while one study used Yanagihara grading scores and local skin temperature change as outcome measure[50].

**Table 1:** Characteristics of included studies.

Study	Sample size	Country	Mean age(yr)	Intervention group	Control group	Primary outcome measurement
Liu 1996	130	China	38.5	A:needles inserted with manual stimulation till elicited de qi; 30min×10 treatment sessions	B(drug group):prednisone 20mg three times /day, vitamin B+ dibazol	Total effective rate
Yu 1999	50	China	39.3	A(acupuncture group):conventional acupuncture 20min/day×10 sessions	B(control group):vitamin B, steriod, traditonal Chinese medicine	Total effective rate
Shao 1999	108	China	combined group: 57; drug-group:56	A(combined group):conventional acupuncture once/day×15d/session, rested for 4 days, another cycle;+ B (drug group)	B(drug group):dexamethasone + vitamin B1 + vitamin B12 + citicotine + ribavirin	Total effective rate
Yang 2001	60	China	electroacupuncture group:39.6±10.5; observed group: 37.8±11.25	A(electroacupuncture group):needles inserted with manual stimulation till elicited de qi; electroacupuncture for 30min/d×10 treatment sessions.	B (observed group): therapeutic apparatus was applied for 2 min/d×10 sessions.	Total effective rate
Zhu 2004	57	China	control group: 45.3±1.3; acupuncture group:44.2±0.8; combined group: 49.1±0.5	A(electroacupuncture group): electroacupuncture 30min/d×13 sessions for a cycle, rested for 2 days, another cycle.+ B(control group)	B(control group):acyclovir, traditional Chinese medicine, physical therapy; C(combined group): A(electroacupuncture group)+B(control group)+stellate ganglion block; rejected, not meet included criteria	Total effective rate
Ma 2004	195	China	control group: 15–44; acupuncture group:16–48	A(acupuncture group):needles inserted with manual stimulation till elicited de qi; 30min/d×5 treatment sessions/week, rested for 2 days, total 6 weeks.	B(control group): vitamin B1 100mg/d×5 times/week, vitamin B12 250ug/d×5 times /week, rested for 2 days, total 6 weeks	Total effective rate
Li 2005	94	China	6–65	A(electroacupuncture group):20min/d×5 days/week, rested for 2 days, a total of 4 weeks	B(control group):Chinese traditional manipulation	Total effective rate
Zhao 2005	42	China	20–72	A(electroacupuncture group): electroacupuncture 20-30min/other day×10 sessions for a cycle.	B(stellate ganglion block group): 10g/L lidocaine 6-8mL, injected at the basal of sixth cervical vertebra transverse process. C(combined group):A (electroacupuncture group) + B(stellate ganglion block group),rejected, not meet included criteria	Yanagihara grading scores; local skin temperature change
Yang 2006	320	China	observed group(n = 106):35.95±11.18; drug group (n = 107):36.79±11.45; acupuncture group (n = 107): 35.95±11.18	A(acupuncture group):horizontal and shallow needles inserted,30min/d×10 treatment sessions, rested for 2 days,2 cycles; B(observed group):A + C	C(drug group): steriod: prednisone oral, 20-40mg daily×4 weeks, descending dose; or dexamethasone IV,5-10mg dail×1–2 weeks then prednisone for 4 weeks; or methylprednisolone IV,100mg daily×5 days then prednisone for 4 weeks; antiviral: aciclovir IV,0.75-1g daily×7-10d;or virazole IV,0.4g daily×7-10d; neurotrophy-medicine: vitamin B+ nicotinic acid	Total effective rate
Zhu 2006	108	China	15–69	A(acupuncture group):shallow needles inserted, 30min/d×5 treatment sessions for acute stage, 30min/d×10 treatment sessions, rested for 2–3 days, 2 cycles for resting stage and restoration stage; B(observed group):A(acupuncture group) + C(drug group)	C(drug group):prednisone 5mg three times/d	Total effective rate
Wang 2007	60	China	therapy group: 15–64, mean 42.2; control group: 18–65, mean 39.5	A(therapy group):needles inserted with manual stimulation till elicited de qi; 30min/d×6 treatment sessions/week, rested for a day, total 2 cycles.+ B(drug group)	B(drug group): dexamethasone 10mg/d ×4d,Vitcmin B12 0.5mg/d +Vitamin B1 100mg×4 days(IM)	H-B grade/2 weeks; Total effective rate
Xuan 2007	126	China	15–65; conventional acupuncture group:48;durg group:46.5	A(conventional acupuncture group):needles inserted with manual stimulation till elicited de qi; 30min×5 treatment sessions/week, 2 weeks for a cycle,+ C. B(horizontal needling group):rejected, not meet included criteria	C(drug group):prednisone 30-60mg/d ×5d, gradually reduced dosage to 5mg/d in 10 days, maintain 5mg/d for 5 days; Vitamin B12 IM,0.5mg/d×10 d.	H-B grade/2 weeks; PSS/2 weeks; ENoG/2 weeks.
Dai 2009	72	China	control group: 27; electro- acupuncture group:29	A(electroacupuncture group): electroacupuncture 20-30min/other day×10 sessions for a cycle.	B(drug group): prednison 1mg/Kg/d ×7d, Vitamin B12 0.5mg/d +Vitamin B1 100mg ×4–5 weeks(IM).	Total effective rate
Tong 2009	119	China	12–95	A:needles inserted with manual stimulation till elicited de qi; 20min×5–10 treatment sessions/ week for inpatients,20min×2–5 treatment sessions/week for outpatients.	B(control group):conventional expectant treatment; C(steroid/drug group): predni- solone 30mg twice daily×1 week+ pepcidine 20mg twice daily×1 week.	H-B grade at 4 weeks; final H-B grade;

### Risk of bias

**Fig 2:**
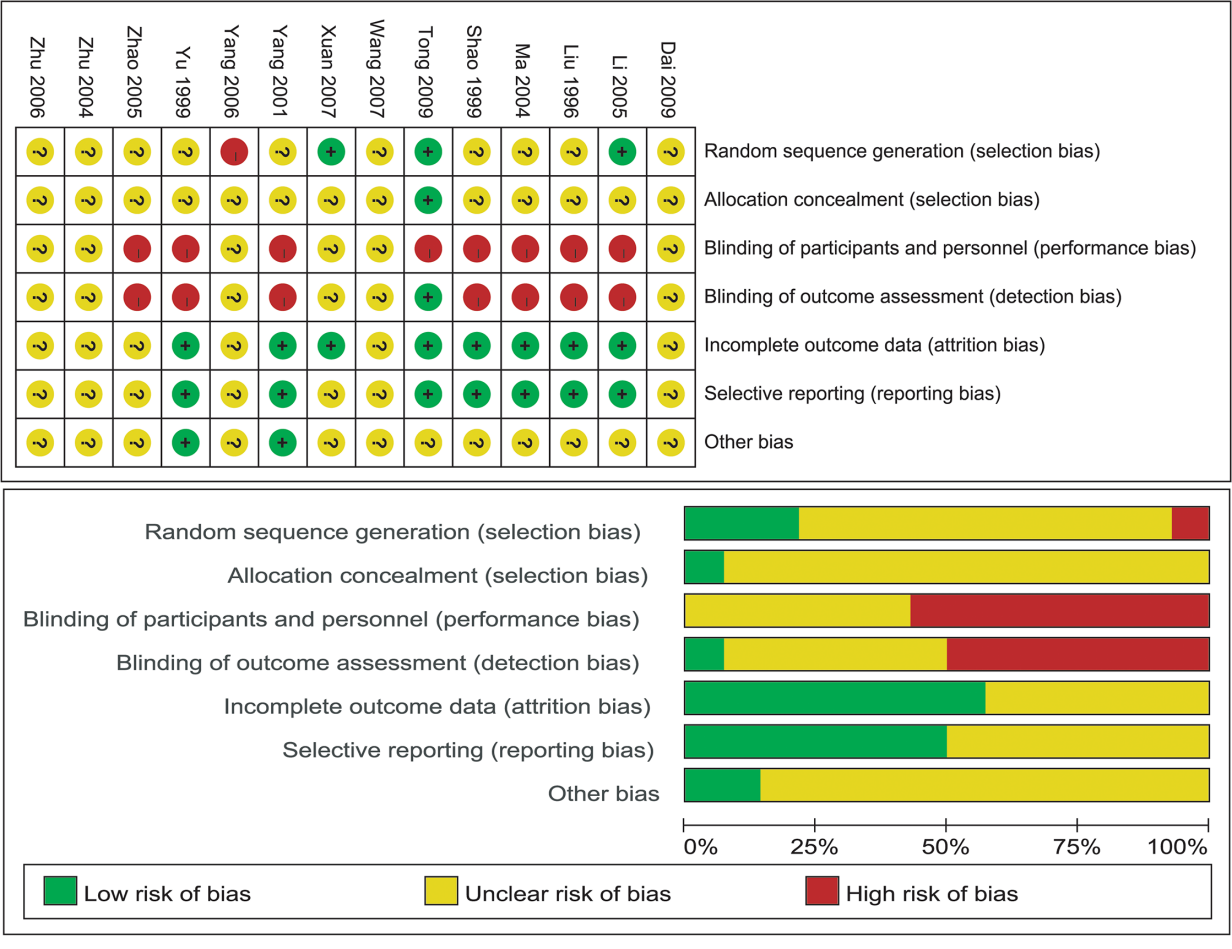
Plots of bias risk.

Plots of risk of bias and methodological quality of included studies are shown in Fig 2. Randomization was performed in all trials; however, only thre studies reported details of random sequence generation[46, 48, 57]. Most of the included studies did not mention allocation concealment, apart from one trial[48]. In most of the included trials, participants and personnel were not blinded. Some studies did not describe the blinding process. All the selected studies were considered at high risk of bias or unclear bias. One studies reported that the outcome assessment was blind[48]. Incomplete outcome data were reported in eight studies, and seven studies conducted selective reporting with a low risk of bias. Only two trials reported other kinds of bias[49, 59].

### Primary outcome: total effective response rate

**Fig 3:**
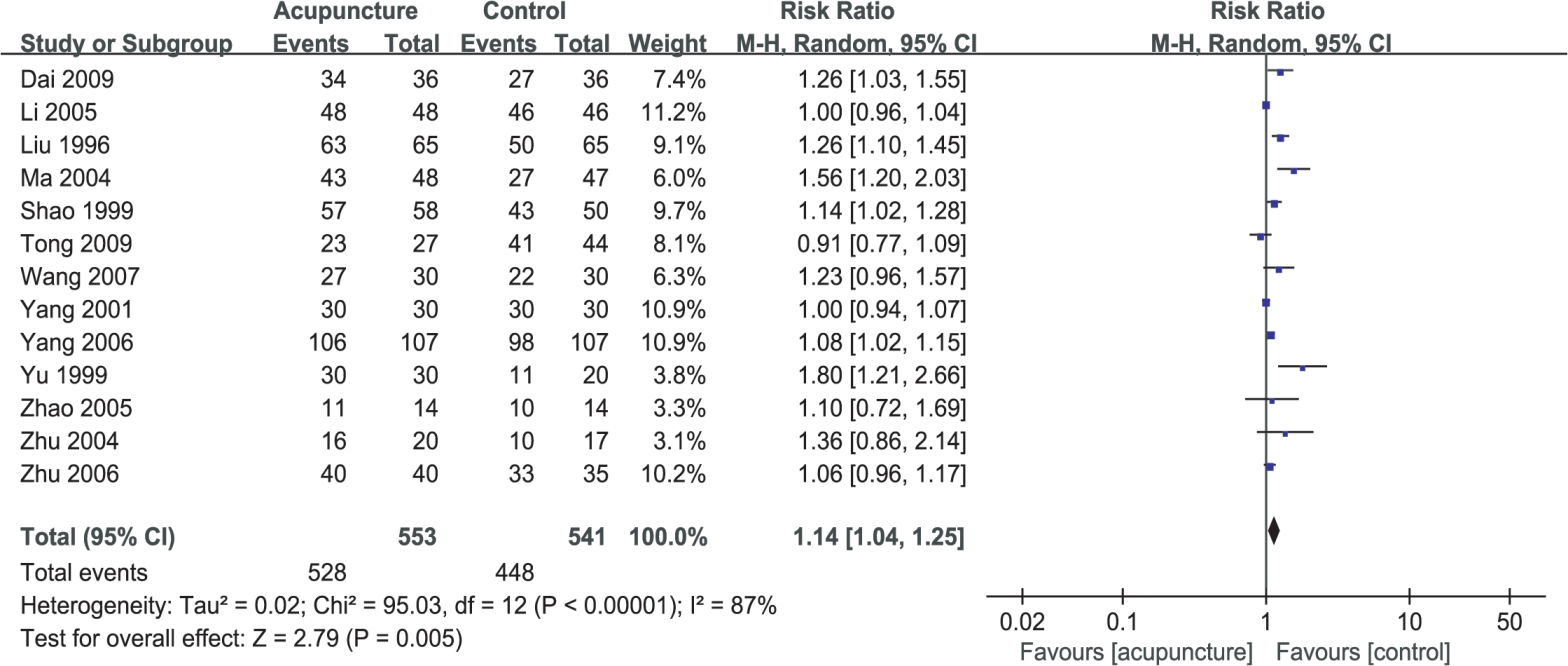
Forest plot of pooled estimate for acupuncture.

A total of 13 RCTs were used to calculate the pooled estimate for assessing the total effective response rate[46–56, 58, 59]. One article[57] could not be combined as it did not use response rate as the outcome measurement. Overall, the total effective response rates in the acupuncture and control groups were 95.48% and 82.81%, respectively. Acupuncture therapy was associated with an increased total effective response rate (RR 1.14, 95% CI: 1.04–1.25, *P* = 0.005, Fig 3), with significant heterogeneity among the included studies (*I* ^2^ = 87%).

### Secondary outcomes

Only two studies reported that no complications occurred during acupuncture[48, 57]. The other studies did not describe side effects or complications. Thus, evaluation of secondary outcomes of complications was not possible.

### Sensitivity analysis

A sensitivity analysis was undertaken based on methodological quality by removing one article at a time sequentially when calculating the pooled effect. However, significant heterogeneity still existed after sensitivity analysis. We speculated that it was probably not a single RCT that contributed to the emergence of heterogeneity, but differences in experimental design, methodology, and other aspects of the articles that might have caused the heterogeneity. Therefore, subgroup analysis was performed to explore the sources of heterogeneity.

### Subgroup analysis

A subgroup analysis was conducted according to experimental design. Eight articles compared acupuncture plus drug therapy against drug therapy in patients with Bell’s palsy and eleven studies compared acupuncture against other interventions. The total effective response rates of these two groups were 96.47% and 96.33%, respectively. Significant associations were observed in both groups (acupuncture plus drug group: RR 1.14, 95% CI 1.08–1.20, *P* < 0.00001; acupuncture group: RR 1.11, 95% CI 1.00–1.24, *P* = 0.05). A total of six studies compared acupuncture against drug therapy, and the result showed significant association in these two groups (RR 1.18, 95% CI 1.02–1.36, *P* = 0.02). No heterogeneity was found in the acupuncture plus drug subgroup (*I* ^2^ = 19%, *P* = 0.29). However, there seems to be huge degree of heterogeneity in the acupuncture versus other intervention subgroup (*I* ^2^ = 90%, *P* < 0.00001) and that versus drug therapy subgroup (*I* ^2^ = 84%, *P* < 0.00001). There are three studies compared acupuncture against blank control. However, the meta-analysis of these three studies is unable to perform because two of these studies’ data is unavailable.

### Publication bias

Egger and Begg tests were conducted to assess publication bias. The results showed no potential publication bias among the included studies (Egger test, *P* = 0.064, 95% CI: −1.080636–3.203404; Begg test, *P* = 0.161).

## Discussion

This is a further systematic review and meta-analysis to assess the efficacy of acupuncture in treating Bell’s palsy, which included 14 RCTs with a total of 1541 participants. This meta-analysis indicated that acupuncture was associated with a greater prevalence of total effective response rate as compared with other therapy interventions in both subgroups. Although a few studies reported that no adverse effects were observed, the overall incidence of side effects and complications of acupuncture was not available for evaluation.

The search result showed that all of the included studies came from China. We feel surprised about this result, and we speculated the possible reasons: 1. Bell’s palsy is kind of self-limited disease, and some patients can be cured without medical treatment. Thus, researches related with Bell’s palsy may be less than other diseases. 2. As a kind of traditional medicine of China, acupuncture treatment is very common in Asian area such as in China and Korea but not so familiar in Europe and USA.

Significant heterogeneity was observed in the included studies overall and in studies that compared acupuncture with other interventions, even when sensitivity analysis and subgroup analysis was performed. Confounding factors, such as experimental design, methodology defects, and operator skills might have contributed to the generation of heterogeneity. It was difficult to find the sources of heterogeneity since methodology shortcomings exist in the included studies. An analysis of bias risk indicated that a high risk of bias was observed in blinding of participants and personnel, and in blinding of outcome assessment.

A previous meta-analysis on the same topic was conducted and published by the Cochrane Library in 2010(17). In detail, this prior meta-analysis included six RCTs, which were also included in our meta-analysis, involving a total of 537 participants. However, no reliable conclusions could be drawn in the Cochrane study, owing to the poor quality of the trials included, in which conventional acupuncture manipulation was compared with other interventions.

Kim *et al*[25]. collected related RCTs on this topic from 15 databases’ inception to December 2010. A total of eight RCTs were enrolled in their study and these trials were also included in our meta-analysis. The quality of these trials was assessed using the Cochrane risk of bias tool and no firm conclusions were made, owing to the low quality of the trials. However, the results of this study are questionable, as the data for one trial were incorrect.

A study conducted by Liu *et al*., one of the included RCTs, compared the efficacy of acupuncture against drug therapy. Each group (the acupuncture and drug groups) comprised 65 patients, while the data in Kim *et al*.’s study were misleading, which may lead to confusing results and conclusions.

In our update meta-analysis, RCTs were found by searching PubMed, Embase, and the Cochrane Central Register of Controlled Trials from inception to July 2014, and more RCTs were included, which may provide more evidence to explore whether acupuncture is beneficial or harmful for Bell’s palsy. Publication bias analyses were also conducted in this meta-analysis.

Cumberworth *et al*[60]. investigated evidence on the topic of acupuncture in patients with Bell’s palsy from 1948 to January 2012. Non-English articles were excluded. This is a review article and meta-analysis was not performed. Cumberworth *et al*. identified three articles with the best evidence: two systematic review articles (Chen *et al*[17]. and Kim *et al*[25].) and one RCT[27]. In this RCT, Ahn *et al*. compared the efficacy of traditional acupuncture against combined acupuncture (traditional plus ear acupuncture). In fact, this RCT was eliminated in our meta-analysis, as studies that compared the effects of different kinds of acupuncture did not meet our inclusion criteria. The authors demonstrated that the efficacy of acupuncture for Bell’s palsy remains unproven.

Teixeira *et al*[61]. performed a systematic review and meta-analysis to evaluate physical therapies for Bell’s palsy in 2011. A total of 12 studies that involved any physical therapy were included, of which five articles[49, 62–65] compared physical therapy with acupuncture. As the authors claimed, these five studies contained no useful data, had a high risk of bias, and no studies reported adverse events. Thus, there was no evidence to prove the efficacy of acupuncture in treating Bell’s palsy. We reviewed these five articles carefully. Three articles[63–65] combined exercise with acupuncture and two[49, 62] compared acupuncture alone. Pan *et al*[62]. conducted a comparison of an acupuncture plus short wave group against an acupuncture group; this was, in fact, a comparison of different kinds of acupuncture, and did not meet our inclusion criteria. A study conducted by Yang *et al*[49]. compared electroacupuncture against therapeutic apparatus, and was included in our meta-analysis.

A systematic review conducted by Lee *et al*[66]. investigated acupuncture trials for Bell’s palsy in 9 databases from the inception to February 2013 in South Korea. However, only the abstract is written English and the text is written in Korean, and we could not read it for further review. As the abstract demonstrated, a total of 10 RCTs were included, but no reliable conclusion could be drawn as to the methodological deficit of the RCTs. From Table 1 in their study, we learned that all included articles comprised at least two combined therapies, which did not meet our inclusion criteria.

Undeniably, although the shortcoming in methodology of the included studies is obvious, the main finding of our meta-analysis further extends previous findings in several aspects. In our meta-analysis, 14 studies comparing conventional acupuncture manipulation or electroacupuncture manipulation with other interventions were included, involving a large sample size of 1541 participants. Eight other published RCTs, were included in our meta-analysis[48, 50–54, 57, 58]. Of the eight additional included trials, only one RCT was high-quality[48], and the other seven showed a high risk of bias. Despite the high risk of bias, acupuncture plus drug therapy was associated with a greater chance of effective response, although heterogeneity existed. As few trials reported side effects and complications, we could not assess the secondary outcome comprehensibly, which is consistent with the previous meta-analysis.

Although acupuncture therapy shows favorable benefits, the effective response rate and recovery may be affected by several factors, such as the learning curve and experience of the manipulator, since acupuncture is a technically difficult and complex process. Subtle differences in the depth and intensity of needles inserted by different operators might also affect the effect of acupuncture treatment, increasing the difficulty of assessing the overall effect of various trials. We believe that operators can reach a unified standard through continued training.

This meta-analysis has some limitations, which are worthy of note. First, almost all studies included were of poor methodological quality, which resulted in a high risk of bias, especially in studies with a small sample size. Second, a huge degree of heterogeneity was observed in the included studies, overall. The experimental design, patients’ characteristics, types of needles used, and degree of operator experience differed, which might have a potential impact on the result of our study. Finally, insufficient information of any side effects or complications of the acupuncture were available, which meant that we were unable to further assess other meaningful clinical endpoints.

Further studies are needed to overcome these limitations and focus on the following aspects. First, experimental design should be improved. Randomization, allocation concealment, and blinding should be performed strictly. Second, the sample size should be enlarged, as overestimation of the overall effect is more likely in small sample size studies compared with large samples. Finally, few of the included studies reported side effects or complications; therefore, further trials should not only focus on the efficacy of acupuncture but should also focus on the safety of acupuncture.

## Conclusions

In summary, the current available evidence is unsufficient to support that acupuncture is an effective therapy for Bell’s palsy due to the poor quality of included researches, and no conclusions can be drawn as to the safety of acupuncture. However, the results should be taken into account cautiously, owing to the poor quality and the heterogeneity of the included studies.

## Supporting Information

S1 Checklist PRISMA Checklist. (DOC)
